# Developmental Changes in PON1 Enzyme Activity in Young Children and Effects of PON1 Polymorphisms

**DOI:** 10.1289/ehp.0900870

**Published:** 2009-06-09

**Authors:** Karen Huen, Kim Harley, Jordan Brooks, Alan Hubbard, Asa Bradman, Brenda Eskenazi, Nina Holland

**Affiliations:** Center for Children’s Environmental Health, School of Public Health, University of California, Berkeley, California, USA

**Keywords:** age, children, enzymatic assay, longitudinal birth cohort, organophosphate metabolism, oxidative stress, paraoxonase, pesticides, PON1 activity

## Abstract

**Background:**

Paraoxonase 1 (PON1) is an enzyme that detoxifies activated organophosphorus pesticides (OPs) and is also involved in oxidative stress pathways.

**Objectives:**

PON1 activity in newborns is lower than in adults, but the ontogeny of PON1 activity is poorly characterized in young children. We examined the effects of age and *PON1* genotype on enzyme activity in a birth cohort of Mexican-American children.

**Methods:**

We determined three substrate-specific measures of PON1 activity in 1,143 plasma samples collected longitudinally from 458 children at five time points from birth through 7 years of age, and genotyped *PON1* polymorphisms at positions 192 and –108 in these children.

**Results:**

Contrary to previous reports that PON1 activities plateau by 2 years of age, we observed an age-dependent increase in all three PON1 measures from birth through 7 years of age (*p* < 0.0001). The *PON1_192_* genotype significantly modified the effect of age on paraoxonase (POase) activity (*p* < 0.0001) such that increases in enzyme activity with age were influenced by the number of R alleles in a dose-dependent manner. Children with the *PON1_-108CC192RR_* diplotype had significantly higher mean PON1 activities and also experienced steeper increases of POase activity over time compared with children with the *PON1_-108TT192QQ_* diplotype.

**Conclusions:**

Lower levels of the PON1 enzyme, which is involved in protection against OPs and oxidative stress, persist in young children past 2 years of age through at least 7 years of age. Future policies addressing pesticide exposure in children should take into account that the window of vulnerability to OPs in young children may last beyond infancy.

The enzyme paraoxonase 1 (PON1; EC 3.1.8.1) detoxifies activated (oxon) organophosphorus pesticides (OPs), which are neurotoxic (Costa et al. 2005a; Furlong et al. 2002). PON1 also is an antioxidant, and several studies indicate that it inhibits oxidation of low-density lipoprotein, a marker of oxidative stress (James 2006; Li et al. 2003). PON1 genotypes and enzyme activities have been associated with numerous health outcomes related to OP exposure and oxidative stress, including Alzheimer’s disease (Erlich et al. 2006), Parkinson’s disease (Zintzaras and Hadjigeorgiou 2004), cardiovascular disease (Bhattacharyya et al. 2008), and preterm birth (Chen et al. 2004; Lawlor et al. 2006). Studies of *in utero* OP exposures have also demonstrated potential gene–environment interactions with PON1. Berkowitz et al. (2004) found that among pregnant women with measureable levels of chlorpyrifos in their blood, those with lower PON1 levels had children with smaller infant head circumferences at birth. The same research group also reported that higher maternal OP exposure (measured by urinary metabolites) was significantly associated with decreased child birth weight among mothers with the *PON1_192QQ_* genotype (Wolff et al. 2007). PON1 enzyme activities and levels vary broadly in humans (Costa et al. 2005b), and the determinants of PON1 variation, including genetics and age, may further explain the role this enzyme plays in relation to exposures and diseases.

Several common polymorphisms in the coding and promoter regions of the *PON1* gene influence substrate-specific PON1 enzyme activities (Brophy et al. 2001; Ferre et al. 2003), particularly single-nucleotide polymorphisms (SNPs) at positions 192 and –108 (James et al. 2005). The SNP at codon 192 results in an amino acid substitution from the active-site residue glutamine (Q) to arginine (R), and the catalytic efficiency of the *PON1_192R_* alloform toward the oxon derivatives of OP pesticides parathion and chlorpyrifos is greater than that of the *PON1_192Q_* alloform. Recent studies have demonstrated that the *PON1_192_* genotype accounts for a large portion of the variation of PON1 activity toward the OP substrate paraoxon in Caucasian and African-American (59%) adults (Bhattacharyya et al. 2008) and also in a Mexican-American (48%) population (Rainwater et al. 2008). Of the promoter polymorphisms, the *PON1_-108_* SNP exerts the most noticeable effect on arylesterase (AREase) activity, a measure of PON1 quantity (22.4%), such that the C allele is associated with increased PON1 levels compared with the T allele (Brophy et al. 2001; Deakin et al. 2003). Although genetic polymorphisms account for a large portion of PON1 variation, it is not sufficient in epidemiologic studies to consider *PON1* genotypes alone (Jarvik et al. 2000; Richter and Furlong 1999). Interindividual variability of PON1 phenotypes can range widely even among individuals with the same *PON1* genotypes because enzyme levels can also be different. Therefore, studies that measure PON1 activities may be more informative than studies that rely solely on *PON1* genotype data.

Several animal studies have demonstrated that low PON1 activity during development results in a reduced metabolic capacity in the young and therefore an increased susceptibility to OP toxicity (Moser et al. 1998; Timchalk et al. 2007). Similarly in humans, PON1 levels in infants are much lower than levels in adults (Chen et al. 2003; Holland et al. 2006). Furthermore, preterm babies have 24% lower activities compared with term babies (Ecobichon and Stephens 1973), suggesting that PON1 levels in the fetus may be even lower. A few studies have measured PON1 activities in healthy young children at single points in time (Garces et al. 2008; Karikas et al. 2006; Sumegova et al. 2007; Vincent-Viry et al. 2003), but only one study has described the ontogenetic profile of PON1 activity in the same individuals over time. This study (Cole et al. 2003) of nine children demonstrated high inter-individual variation of PON1 from birth to 2 years of age; PON1 activities increased over time and reached a plateau between 6 months and 2 years of age in most, but not all, individuals. PON1 activity is relatively stable in adults (Costa et al. 2005b); however, in the elderly, it decreases steadily over time (Seres et al. 2004). To date, longitudinal changes in PON1 activity in children after 2 years of age and potential differences between genotypes have not yet been characterized.

Previously, we reported a relatively high level of OP exposure in the Salinas Valley, California, the location of our longitudinal birth cohort (Bradman et al. 2005, 2007). Additionally, we observed associations between *in utero* OP exposure and decreases in gestational duration (Eskenazi et al. 2004), abnormal reflexes in neonates (Young et al. 2005), poorer mental development at 2 years of age (Eskenazi et al. 2008), and increases in maternal report of pervasive development disorder (Eskenazi et al. 2008) in these children. Initially, we reported PON1 activity in 130 mothers and their newborns and demonstrated much lower activities in the newborns (Furlong et al. 2006; Holland et al. 2006). In the present study, we measured PON1 activity in 458 children at several time points spanning birth through 7 years of age to determine the ontogeny of PON1 phenotypes. In the same children, we also genotyped two *PON1* SNPs (–108 and 192) known to influence phenotypic variation and examined how *PON1* genotypes affect children’s PON1 activities at different ages.

## Materials and Methods

### Study subjects

The Center for Health Assessment in Mothers and Children of Salinas (CHAMACOS) study is a longitudinal birth cohort study of the effects of pesticide and other environmental exposures on neurodevelopment, growth, and respiratory disease in children from primarily Mexican-American families (Eskenazi et al. 2003). Located in Monterey County, California, the Salinas Valley is an area of heavy agricultural production where approximately 200,000 kg of OPs are applied annually (Department of Pesticide Regulation 2005). Women and children in this community are at risk for higher exposures to pesticides through direct exposure if they live near agricultural fields or if the mother works in the field, and through the take-home pathway. A total of 601 pregnant women were enrolled in the CHAMACOS study, and 528 delivered newborns. Mothers in the CHAMACOS cohort were primarily young (mean ± SD, 25.6 ± 5.3 years), married, low-income, Mexican-born, Spanish-speaking women who were farmworkers themselves (44%) or lived with farmworkers at the time of enrollment (84%). Ethnicity of children and mothers was based on mothers’ self-report. For this particular study, we limited analyses to include only subjects who were of Mexican descent (97%). More than half the women in CHAMACOS came from three states in Mexico: Michoacán (23%), Guanajuato (21%), and Jalisco (11%). Study protocols were approved by the University of California–Berkeley human subjects review committee. Written informed consent was obtained from all mothers, which included consent for their children to participate.

### Blood collection and processing

Blood specimens were collected from 458 CHAMACOS children (50.1% boys) for the measurement of PON1 enzyme activity. Umbilical cord blood samples (*n* = 336) were collected by delivery room staff once the baby was safely delivered. Blood specimens were also collected from children at approximately 1 year of age (*n* = 52; mean ± SD, 1.07 ± 0.19 years), 2 years of age (*n* = 249; 2.01 ± 0.09 years), 5 years of age (*n* = 225; 5.10 ± 0.22 years), and 7 years of age (*n* = 281; 7.10 ± 0.23 years). We were able to measure PON1 enzyme activity at four or more time points for 108 of these children.

Heparinized whole blood was collected in BD Vacutainers (Becton, Dickinson and Company, Franklin Lakes, NJ), centrifuged, and then divided into plasma, buffy coats, and red blood cells and stored at −80°C. Vacutainers without anticoagulant were used to collect serum and clot. DNA was isolated from blood clots as described previously (Holland et al. 2006).

### Determination of PON1 levels and activities

We measured PON1 enzyme activity toward three substrates [paraoxon (PO), phenyl acetate (aryl ester; ARE), and chlorpyrifos-oxon (CPO)] in plasma samples using spectrophotometric methods as described previously (Richter and Furlong 1999). The AREase assay is considered a measure of PON1 enzyme quantity because rates of phenyl acetate hydrolysis do not differ between *PON1_192_* Q and R alloforms as they do for paraoxon hydrolysis (POase). Methods based on ELISA and Western blot using PON1 antibodies confirm a high correlation (*r* > 0.85) between measured PON1 quantity and AREase activity (Connelly et al. 2008; Kujiraoka et al. 2000). In contrast, POase and chlorpyrifos-oxonase (CPOase) substrate-specific assays reflect both quantity and catalytic efficiency of the enzyme. In this article, we refer to enzyme quantity measured by AREase activity as PON1 levels. CPOase and POase activities (quantity and substrate-specific efficiency) are referred to as enzyme activity. All assays were performed in triplicate. Quality assurance, described in more detail by Huen et al. (2009), included assessment of repeat samples (separate aliquots of the same sample run on different days), internal controls (aliquots of the same sample run on all assay plates), and concurrent analyses of specimens from different time points (samples from different time points run on the same plates). Repeated analysis of 3% of samples showed a high degree of concordance. For the three substrate-specific assays, the average coefficient of variation (CV) for repeated samples ranged from 6% to 9%, and the correlation coefficients between repeated runs ranged from 0.91 to 0.98. Interassay variability, as measured by the average CV for internal controls samples, was between 7 and 9%.

### Determination of PON1 genotypes

The coding polymorphism, *PON1_192_*, was genotyped using the TaqMan real-time polymerase chain reaction (PCR) method. Briefly, primers for the nucleotide sequence flanking the SNP and probes specific for the SNP were custom designed by Applied Biosystems, Inc. (Foster City, CA). The promoter SNP, *PON1_-108_*, was genotyped using a fluorogenic allele-specific genotyping assay (Amplifluor; Flowgen Biosciences Ltd., Nottingham, UK). This assay required a two-part nested PCR strategy, where the region surrounding the SNP was preamplified using nonallelic flanking primers. The amplicon was then diluted and used as the template for the Amplifluor assay. Quality assurance procedures for genotyping both *PON1* SNPs included assessment of randomly distributed blank samples in each plate and duplicates of randomly selected samples with independently isolated DNA from the same subjects. Repeated analysis (4% of samples) in several runs showed a high degree (> 99%) of concordance. All discrepancies were resolved with additional genotyping.

### Statistical analysis

To model longitudinal changes of PON1 enzyme activity, we used generalized estimating equations (GEEs) with an exchangeable correlation structure because we had repeated measures on individual subjects. This method is often advantageous in longitudinal models because inferences based on robust estimators are less dependent on the assumption that the data are normally distributed (Hox 2002). Correlations of measurements on individuals at various ages were similar. We created separate models for each of the three outcomes, AREase, CPOase, and POase activity. Independent variables in the model included age (years), *PON1* genotype (either *PON1_192_* or *PON1_-108_*), and an inter-action term between age and *PON1* genotype (age × *PON1_192_* or age × *PON1_-108_*). Because the number of specimens collected at 1 year of age was much smaller than at other time points, we also ran GEE models without this time point. However, because results did not change significantly, we show only the GEE models using all time points.

We previously established that storage duration can affect PON1 activity, particularly in specimens that had been stored for > 2 years (Huen et al. 2009). PON1 activity decreased on average by 17.1%, 39.4%, and 37.6% for AREase, CPOase, and POase, respectively after 5 years of storage (*p* < 0.001 for all three assays). This decline of PON1 enzymatic activity over time has also been reported in other studies (Brackley et al. 1983; Stenzel et al. 2007). In this study, specimens collected at earlier ages were sometimes stored for > 2 years, so age and storage duration were inversely correlated with each other (Pearson’s correlation coefficient *r* = 0.986). To estimate the effects of age while taking into account the potential effects of storage duration, we generated a storage duration correction factor using pilot data from a previous study (Huen et al. 2009). Briefly, parallel aliquots of specimens from 95 subjects were assayed after 2 years and 7 years of storage at −80°C. Measured AREase, CPOase, and POase activities were significantly lower after 7 years of storage compared with 2 years of storage. Using these data, we constructed linear models to predict the percent change in PON1 activity as a function of years of storage duration and storage duration × assay temperature. The models including an interaction between storage duration and assay temperature had the lowest Akaike Information Criterion and were thus selected to be used to determine the correction factors. The intercept was constrained to equal zero. We then used the resultant coefficients from these models as correction factors to predict the PON1 activity (AREase, CPOase, POase) at a storage time of zero years, and used the storage duration–corrected PON1 activity measures in subsequent GEE models to determine the effects of age on PON1 activity as described above.

Because we could not completely eliminate the residual confounding by storage duration in our GEE models, we conducted additional analyses to provide evidence that the association between age and PON1 substrate-specific measures is not attributable solely to the strong correlation of age with storage duration. We performed a likelihood ratio test comparing a full model, including terms for age, storage, PON1 genotype, and the interaction between age and genotype, with a restricted model without the age terms. It indicated that age should be retained in the model and that the fit of the full model was significantly better (*p* < 0.0001). Additionally, because the correlation between age and storage duration was considerably weaker within shorter time intervals (*r* = −0.3 between 1- and 2-year-olds and *r* = −0.93 between 5- and 7-year-olds), we also ran GEE models including a term for duration of storage in data sets restricted *a*) only to 1- and 2-year-olds and *b*) only to 5- and 7-year-olds. In the models for 1- and 2-year-olds, an interaction term for PON1 genotype and age was not included because there was not sufficient power with the limited sample size.

All analyses were performed in STATA (version 10.0; StataCorp, College Station, TX) and R (version 2.7.0; R Development Core Team 2008).

## Results

### PON1 polymorphisms

Genotype distributions did not deviate significantly from Hardy–Weinberg equilibrium. For the *PON1_192_* polymorphism, the allele frequencies of the Q and R alleles were 50.2% and 49.8%, respectively, and genotype frequencies were 23.2%, 53.1%, and 23.7% for QQ, QR, and RR genotypes (Table 1), respectively. Genotype frequencies for *PON1_-108_* were 28.3%, 53.3%, and 18.4% for CC, CT, and TT, respectively. The allele frequency of the major allele C was 54.7%.

### Effect of age on enzyme activity

Table 2 lists descriptive statistics for AREase, CPOase, and POase activity at birth and 1, 2, 5, and 7 years of age. Mean PON1 enzyme activity in CHAMACOS children was lowest in newborns for all three substrate-specific assays and highest at 7 years of age, and there were no differences in PON1 activity by sex (analysis of variance, *F* < 1.14; *p* > 0.30 for all three PON1 assays at all time points). Mean ± SD AREase activity, which is considered a measure of PON1 levels (quantity), approximately doubled from 33.1 ± 14.5 U/mL at birth to 66.5 ± 30.4 U/mL at 1 year of age and quadrupled (compared with birth) to 121.5 ± 30.3 U/mL at 7 years of age. Although PON1 activity did not increase appreciably between 2 and 5 years of age for AREase and CPOase, all three substrate specific measures grew noticeably between 5 and 7 years of age. At birth, differences in overall enzymatic activity between newborns with the lowest and highest values were 26-, 34-, and 136-fold for AREase, CPOase, and POase, respectively. In 7-year-old children, the fold differences were somewhat smaller (17-, 13-, and 21-fold for AREase, CPOase, and POase, respectively).

### Effects of PON1 genotype on enzyme activity

**Figure 1:**
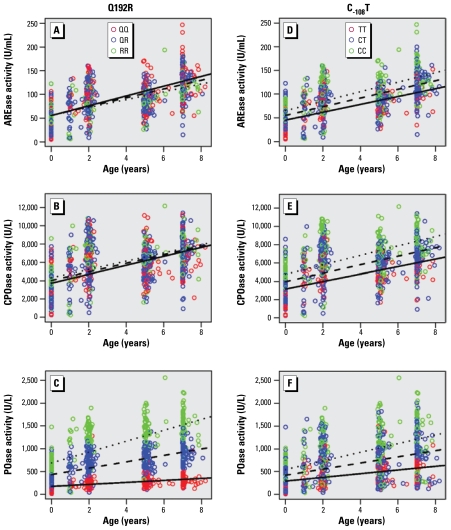
PON1 enzyme activity by age (years) and PON1 genotype. (*A*–*C*) AREase (*A*), CPOase (*B*), and POase (*C*) activity (corrected for storage duration) plotted by age for QQ (red), QR (blue), and RR (green) PON1_192_ genotypes. (*D*–*F*) PON1 enzyme activities plotted by age for CC (green), CT (blue), and TT (red) PON1_-108_ genotypes. The overlaid regression lines are the predicted values generated by GEE models on the storage duration–corrected data as shown in Table 4. For all three substrate-specific assays, PON1 activity increased with age (*p* < 0.0001). The effect of age on POase activity significantly differed by genotype, as illustrated by the steeper slopes for RR (119.2 U/L per year; 95% CI, 97.7–140.9) children compared with QQ (21.8 U/L per year; 95% CI, 15.3–28.3) children (*C*). The C_-108_T genotype was significantly associated with all three assays (*D*–*F*). CC children had the highest mean activities, whereas TT children had the lowest mean activities. For POase activity, the slopes for CC children were steeper than slopes for CT and TT children (*F*), demonstrating the significant interaction between age and C_-108_T genotype (*p*< 0.0001).

Within each age group, the PON1 enzyme levels were influenced by both *PON1_192_* and *PON1_-108_*. Mean AREase values differed by the *PON1_192_* polymorphism (*p* for trend < 0.05 for increasing number of R alleles). At birth, average AREase levels were 15.8% higher in RR children than QQ children (Table 1). In contrast, at 2, 5, and 7 years of age, mean AREase activity was 9.8–14.1% higher in QQ children than in RR children. Mean AREase activity also varied by *PON1_-108_* genotypes, and these differences were larger at all ages than for *PON1_192_*. At birth, mean AREase activity was 18.9 U/mL or about 44.9% lower in TT newborns than in CC newborns (Figure 1D). PON1 levels continued to be lower in CC children (*p* for trend < 0.001 for all time points except at year 1), with differences ranging from 18.8% to 25.4% in 2-, 5-, and 7-year-old children.

The *PON1_192_* SNP explained a relatively small amount of the variance of AREase activity at different ages (1.4–3.6%), whereas the promoter polymorphism *PON1_-108_* accounted for 8.2–24.4% of AREase activity [see Supplemental Material, Table 1 (available online at doi:10.1289/ehp.0900870.S1 via http://dx.doi.org)]. Although 4.4% of the variance of CPOase activity was explained by the *PON1_192_* SNP at birth, the coding region SNP made a seemingly smaller contribution at other time points (0.1–1.2%), and the role of *PON1_-108_* seemed much larger (4.1–27.3%). Both SNPs accounted for a significant portion of POase activity variation. The contribution of *PON1_192_*, which was 39.8% at birth, was generally higher in older children and highest at 7 years of age (70.7%).

### Effects of age and PON1 genotype on enzyme activity

We used GEE augmentation of linear models to determine the effects of age and genotype on PON1 activity in children (Table 3). Point estimates in the models using storage duration–corrected data were not markedly different from those generated using raw data, although 95% confidence intervals (CIs) were slightly wider in storage duration–corrected models. Because the results for both models were similar, only results from the storage duration–corrected models are shown. Regression lines were generated for AREase, CPOase, and POase activity by age and are plotted with respect to *PON1_192_* (Figure 1A–C) and *PON1_-108_* (Figure 1D–F) genotypes. All three substrate-specific PON1 enzyme activities were positively associated with age (*p* < 0.0001 for all models). It appeared that PON1 activities continued to increase past the age of five because paired *t*-tests showed that AREase, CPOase, and POase were all significantly higher in 7-year-olds than in 5-year-olds (*p* < 0.0005).

For AREase activity, there was a small but statistically significant interaction (*p* = 0.016) between age and *PON1_192_* genotype in which the slopes for age were lowest (8.3 U/mL per year) for RR children and highest (10.3 U/mL per year) for QQ children (Figure 1A). The promoter polymorphism, *PON1_-108_*, had a more pronounced effect on AREase activity than did *PON1_192_*. Furthermore, we observed a small but significant interaction (*p* = 0.027) between age and *PON1_-108_* genotype in which mean AREase activity increased 10.0, 9.1, and 8.2 U/mL per year increase in age for CC, CT, and TT children, respectively.

Similar to AREase, CPOase activity was modestly associated (*p* = 0.017) with *PON1_192_* genotype (Table 4, Figure 1B). Mean CPOase activity was 591.0 U/mL (95% CI, 104.6–1,077.4 U/mL) higher in RR children and 295.5 U/mL (95% CI, 52.3–538.7 U/mL) higher in QR children than in QQ children. There was no interaction between age and *PON1_192_* genotype. In contrast, we did observe a small but significant interaction between age and *PON1_-108_* genotype (β-estimate for age × *PON1_-108_* genotype = −54.6, *p* = 0.022).

We also observed significant effect modification by the *PON1_192_* genotype (*p* < 0.0001) on the relationship between age and POase activity (Figure 1C). POase activity increased with age for all *PON1_192_* genotypes, but the slopes for age were much steeper for RR children. For example, mean activity increased by only 21.8 U/L (95% CI, 15.3–28.3 U/L) per year in QQ children, whereas in RR children mean activity increased 119.2 U/L or almost 5.5-fold more per year (95% CI, 97.7–140.9 U/L). The effect is so large that even at 7 years of age the average POase activity in QQ children was lower than the average POase activity in QR and RR children at 2 years of age. Although not as strikingly as the coding polymorphism *PON1_192_*, the promoter SNP, *PON1_-108_*, also influenced POase activity significantly (Figure 1F). The effect of age on POase activity was modified by *PON1_-108_* genotype (*p* < 0.0001) such that slopes for mean POase activity per year were highest in CC children and lowest in TT children.

To determine the effects of combinations of *PON1_192_* and *PON1_-108_* genotypes on enzyme activity, we performed GEE models comparing children with the *PON1_-108CC192RR_* homozygous diplotype (reference group) with those with the *PON1_-108TT192QQ_* homozygous diplotype (Table 4). Children with the *PON1_-108TT192QQ_* diplotype had significantly lower mean AREase (14.1 U/mL; 95% CI, 3.7–24.4 U/mL), CPOase (1525.9 U/L; 95% CI, 835.0–2216.3 U/L), and POase (627.4 U/L; 95% CI, 534.5–720.4 U/L) activities compared with *PON1_-108CC192RR_* children. Additionally, we observed a statistically significant interaction between age and diplotype on POase activity (*p* < 0.0001). The slope for mean POase activity over age was much steeper for *PON1_-108CC192RR_* children (135.3 U/L per year; 95% CI, 115.0–155.7 U/L) than for *PON1_-108TT192QQ_* children, which was 112.6 U/L per year (95% CI, 91.3–133.8 U/L) lower.

### Effects of age and PON1 genotype on enzyme activity in restricted data sets

Because the correlation between age and storage duration was appreciably smaller within shorter time intervals, we were able to include a storage duration term (rather than use the storage duration–corrected data) in GEE models where the time interval was restricted to *a*) 1- and 2-year-olds only or *b*) 5- and 7-year-olds only. The results are presented in Table 4. For 1- and 2-year-olds, age was significantly associated with all three substrate-specific measures of PON1 even after adjusting for potential cofounding by storage duration (*p* < 0.001 for AREase and CPOase and *p* = 0.016 for POase). Further, storage duration was only significantly associated with POase activity (*p* = 0.04), and the magnitude of the effect (−55.8 U/L) was much smaller than the effect of age (128.0 U/L). Similarly, for 5- and 7-year-olds, age was strongly associated with AREase, CPOase, and POase (*p* < 0.001) activities, and storage duration was not. For POase, the prominent interaction between age and PON1_192_ genotype observed in the storage duration–corrected models was also significant in these models (*p* = 0.02).

### Correlation analysis

Correlations of each of the PON1 substrate-specific enzyme activities in children at different time points were highly significant except at 1 year of age, when there was a weak correlation with 7-year-olds and no correlation with cord blood samples at birth. This may be attributable to the relatively small sample size at 1 year of age. Further, because the cord blood measurement accounts for both fetal and placental expression, the correlation between these two time points (birth and 1 year of age) may be weaker than with other age groups. For pairwise comparisons among all other age groups (birth and 2, 5, and 7 years of age), Pearson’s correlation coefficients ranged from 0.37 to 0.62 for AREase and from 0.41 to 0.73 for CPOase assays. POase measurements were even more highly correlated, with correlation coefficients ranging from 0.70 to 0.93 [see Supplemental Material, Table 2 (available online at doi:10.1289/ehp.0900870.S1)]. Within each time point, all three PON1 activities were also significantly correlated with each other (*p* < 0.001). The correlation coefficients tended to be highest between CPOase and AREase, ranging from 0.84 to 0.97. The pairwise correlation coefficients between POase and CPOase ranged from 0.44 to 0.78. Between POase and AREase, the correlation coefficients were lower, ranging from 0.23 to 0.65 [see Supplemental Material, Table 3 (available online at doi:10.1289/ehp.0900870.S1)].

## Discussion

In this study, we examined the ontogeny of PON1 phenotypes in a longitudinal birth cohort of 458 children who were followed from birth through 7 years of age. Our data demonstrated a dramatic range of PON1 variability in young children, particularly in newborns. We observed an age-dependent increase in measures of PON1 levels and enzymatic activity that continued through 7 years of age. Furthermore, for POase activity, the rates of increase were allele specific, such that children with *PON1_192R_* alleles experienced a much more pronounced increase in POase activity over time. Similarly, children with the *PON1_-108CC192RR_* diplotype had significantly higher mean PON1 activities and also experienced steeper increases of POase activity over time compared with children with the *PON1_-108TT192QQ_* diplotype.

Earlier studies (Chen et al. 2003; Holland et al. 2006 ) demonstrated that newborns have lower PON1 levels than adults. Cole et al. (2003) reported that PON1 levels (AREase) and activities (POase) increased noticeably in nine children during the first year of life and appeared to have plateaued by 2 years of age in some but not all of them. In contrast, our study, which followed a much larger number of children for a longer period of time, demonstrated that children’s PON1 levels and activities continue to increase past 2 years of age until at least 7 years of age. These data suggest that children’s PON1 activities remain lower than their expected adult levels longer than previously thought. Although this phenomenon has not yet been reported in other epidemiologic studies, similar observations in mice have also shown that PON1 levels are low at birth and increase significantly with age (Zemke et al. 2009). Because PON1 can detoxify activated OPs and also has antioxidant properties, our findings provide evidence that the time in which infants and young children remain more vulnerable than adults to OP exposures and oxidative stress may extend from birth through 7 years of age and possibly even later.

In addition to the age-dependent increases in PON1 measures, our data in this large Mexican-American cohort corroborate allele and genotype frequencies of the *PON1* SNPs at positions –108 and 192 in other studies of Mexican and Mexican-American populations (Rainwater et al. 2008; Rojas-Garcia et al. 2005). Consistent with other studies, we found that the Q allele of the *PON1_192_* coding polymorphism was associated with decreased POase activities and that the C allele of the *PON1_-108_* promoter polymorphism was associated with increased activity in all three PON1 measures (Deakin and James 2004; Furlong et al. 2005). In transgenic mouse models, the *PON1_192Q_* alloform does not protect against CPO exposure as well as the *PON1_192R_* alloform (Cole et al. 2005). Furthermore, mice with lower PON1 levels were more sensitive to OP toxicity as measured by cholinesterase inhibition in the brain (Li et al. 2000). These studies demonstrated that because of their influence on PON1 levels and catalytic efficiency, the *PON1_-108_* and *PON1_192_* genotypes are significant determinants of sensitivity to OP exposure. Furlong et al. (2006) used these data to estimate that the range of sensitivity to CPO in CHAMACOS mothers and their newborns is very wide (131- to 164-fold). Although age and PON1 genotype appear to affect this variability significantly, it is likely that other factors such as environmental exposures and additional genetic polymorphisms may account for some of this variability.

To our knowledge, no other studies have examined the effects of common polymorphisms on the ontogeny of PON1 phenotypes in young children. In our study, children with the *PON1_192R_* and *PON1_-108C_* alleles experienced much steeper increases in POase activity with age in a dose-dependent manner (dose of allele), indicating significant effect modification by *PON1_192_* and *PON1_-108_* genotypes. A similar pattern was reported by Parker-Katiraee et al. (2008), who found that PON1 expression in murine liver tissue increased during embryonic development in an allele-specific manner. These data suggest that PON1 ontogeny is influenced by genetic polymorphisms and children with certain genotypes (*PON1_192Q_* and *PON1_-108T_* alleles) may be more susceptible to the adverse effects of OP exposure and oxidative stress throughout childhood. In addition to PON1 genotypes, PON1 developmental expression may also be affected by prenatal exposure to OPs via epigenetic mechanisms. It will be interesting to determine whether OP exposure may be associated with PON1 ontogeny in future studies.

In our analyses, we performed GEE models using raw data and also using data corrected for storage duration. Previous studies, including our own, have reported a decline in POase, AREase, and diazoxonase activities over time, particularly in samples stored for several years (Brackley et al. 1983; Huen et al. 2009; Stenzel et al. 2007). To adjust for storage duration, we created a correction factor by constructing linear models from a data set of 95 subjects whose PON1 activities were measured at two time points. Although it is possible that the relationship between storage duration and PON1 activity may not be linear, it was the most reasonable assumption to use with only two time points available. This assumption would tend to overestimate the effect of storage duration, thus biasing the effect of age on PON1 activity toward the null. We also assumed that variance in measurements at the two time points was due primarily to storage duration and not other unmeasured factors. Although the storage duration–corrected data introduces a degree of uncertainty into our models, we were still able to identify highly significant age-dependent increases in all three PON1 measures using this data. In addition to the models using storage duration data, we were able to apply GEE models to subsets of the data (smaller time intervals) in which the correlation between age and storage duration was weaker. In these models, where a term for storage duration was included, we confirmed our findings that age is positively associated with PON1 activity after adjustment for storage duration. Despite the potential limitations of our analysis, this is the largest longitudinal study examining PON1 activity during childhood development. Use of a cross-sectional study design in future studies may help to confirm our results while avoiding the potential bias introduced by storage duration effects on PON1 activity.

From our data, it is not yet apparent when children’s PON1 levels and activities reach mature levels. Furthermore, levels did not increase appreciably between 2 and 5 years of age, but then continued to increase between 5 and 7 years of age. It is possible that the approach to puberty may affect levels of this physiologically important enzyme. Compared with younger children, average PON1 levels and activities in 7-year-olds were much closer to those we previously reported in pregnant CHAMACOS mothers (Holland et al. 2006). Whether children with different genotypes reach mature levels at different ages remains unclear. Because this study was performed in a cohort of Mexican-American children with relatively high levels of OP exposure, generalization of the age-dependent increase of PON1 activity to other populations should be done with caution. Future studies of PON1 ontogeny should include children as they grow older (past 7 years of age) and also additional populations.

Our present study provides additional evidence that children with the *PON1_192QQ_* and *PON1_-108TT_* genotypes may represent vulnerable populations. This may be of particular concern in the Mexican-American population, including our CHAMACOS subjects, because both genotypes are relatively common (23.2% QQ, 18.4% TT, and 7.5% QQ and TT). The *PON1_192Q_* and *PON1_-108T_* alleles are also quite frequent in Caucasian populations (73% Q, 62% T) but much less so in African-American populations (27% Q, 15% T) (Chen et al. 2003). Thus, sensitivity to OP toxicity and oxidative stress may vary between populations.

In summary, we followed a cohort of Mexican-American children from a farm-worker community from birth through 7 years of age and found that PON1 levels and activities continue to increase past 2 years of age and possibly even past 7 years of age. Additionally, *PON1* polymorphisms modify the effect of age on enzyme activity such that some children experience much steeper increases over time than others. These findings suggest that children’s PON1 activities continue to increase until much later than previously believed, and therefore, the window of increased susceptibility to OP exposures and oxidative stress may also last much longer in young children. Future policies regarding protection from pesticide exposures should take into account that children may be particularly vulnerable to OP exposures and those with certain PON1 genotypes may be at even greater risk because of lower PON1 levels and activities.

## Figures and Tables

**Table 1:** *PON1* polymorphisms and AREase (U/mL) enzyme activity in children of various age groups.

	Frequency^a^ (%)	Birth (*n* = 36)		1 year (*n* = 52)		2 years (*n* = 249)		5 years (*n* = 225)		7 years (*n* = 281)		Mothers^b^ (*n* = 130)	
		Mean ± SD	Range	Mean ± SD	Range	Mean ± SD	Range	Mean ± SD	Range	Mean ± SD	Range	Mean ± SD	Range
192													
QQ	23.2	31.7 ± 15.5*	3.9–70.9	77.5 ± 22.7	52.2–126.6	93.0 ± 25.2*	16.4–143.3	92.3 ± 26.9*	36.5–157.9	129.5 ± 36.3	52.9–246.3	151.9 ± 46.8	19.8–237.5
QR	53.1	32.2 ± 14.4	3.8–98.0	65.6 ± 29.6	7.3–125.4	84.8 ± 25.8	8.5–151.2	81.5 ± 20.5	22.4–131.3	119.8 ± 29.1	14.5–184.6	144.3 ± 44.6	72.9–261.9
RR	23.7	36.7 ± 13.0	9.7–73.6	59.5 ± 36.3	5.9–103.5	84.7 ± 23.0	17.7–136.9	80.9 ± 18.6	22.9–111.3	117.6 ± 25.3	61.1–190.4	152.2 ± 48.6	78.9–281.4
–108													
CC	28.3	42.1 ± 13.0**	13.9–90.3	73.8 ± 34.9	5.9–125.4	98.2 ± 27.7**	13.9–151.2	95.8 ± 23.8**	42.0–157.9	138.5 ± 30.3**	73.3–246.3	163.6 ± 51.0*	78.9–281.4
CT	53.3	32.0 ± 13.5	3.8–98.0	64.8 ± 29.5	7.3–126.6	83.7 ± 22.8	8.5–143.3	81.3 ± 19.4	22.9–144.0	120.6 ± 27.6	14.5–198.9	147.1 ± 43.3	19.8–242.8
TT	18.4	23.2 ± 11.1	3.9–54.7	59.0 ± 27.6	10.0–99.3	79.7 ± 21.8	26.7–137.6	73.9 ± 20.4	22.4–109.8	103.3 ± 23.7	33.6–159.9	134.8 ± 43.3	54.5–233.7

a Genotype frequency was reported for children with enzyme activity measured at birth. Other time points had similar frequencies.

b Measurements of PON1 enzymatic activity were taken in pregnant mothers at 26 weeks’ gestation as described previously (Holland et al. 2006).

* *p* < 0.05,

** *p* < 0.001, determined by Cuzick’s test for trend.

**Table 2:** Summary of PON1 enzymatic activity in children and mothers.

		AREase (U/mL)		CPOase (U/L)		POase (U/L)	
Age (years)	No.	Mean ± SD	Range	Mean ± SD	Range	Mean ± SD	Range
0	336	33.1 ± 14.5	3.8–98.0	2020.2 ± 860.7	153.9–5255.2	257.1 ± 162.4	7.5–1017.5
1	52	66.5 ± 30.4	5.8–126.6	3995.1 ± 1885.7	195.2–7667.0	570.0 ± 417.1	37.3–1502.7
2	249	87.0 ± 25.1	8.5–151.2	5310.3 ± 1549.9	408.7–9474.5	667.3 ± 390.5	37.5–1813.1
5	225	84.0 ± 22.3	22.4–157.9	5502.4 ± 1424.1	1433.0–9808.1	732.0 ± 417.1	97.6–1855.6
7	281	121.5 ± 30.3	14.5–246.3	7035.8 ± 1700.6	904.2–11576.7	894.2 ± 477.0	119.5–2504.5
Mothers^a^	130	149.2 ± 46.1	19.8–281.4	9358.3 ± 2794.4	1661.7–17098.0	1024.2 ± 656.4	66.1–3014.2

a Measurements of PON1 enzymatic activity were taken in pregnant mothers at 26 weeks’ gestation as described previously (Holland et al. 2006).

**Table 3:** Regression analysis (GEE) of the effects of age and PON1 genotype on PON1 enzyme activity (storage duration–corrected data).

	Q-_192_R^a^			C-_108_T^b^			Q-_192_R and C-_108_T homozygous diplotypes^c^		
	β-Estimate	95% CI	*p*-Value	β-Estimate	95% CI	*p*-Value	β-Estimate	95% CI	*p*-Value
AREase									
Intercept	55.3	50.8 to 59.8	< 0.0001	64.8	61.0 to 68.7	< 0.0001	64.5	58.6 to 70.3	< 0.0001
Age^d^	10.3	9.2 to 11.4	< 0.0001	10.0	9.1 to 11.0	< 0.0001	9.0	7.8 to 10.3	< 0.0001
Genotype	0.3	−3.3 to 3.8	0.884	−9.9	−13.3 to −6.4	< 0.0001	−14.1	−24.4 to −3.7	0.008
Age × genotype^e^	−1.0	−1.9 to −0.2	0.016	−0.9	−1.7 to −0.1	0.027	0.04	−0.1 to 0.2	0.648

CPOase									
Intercept	3728.1	3431.1 to 4025.1	< 0.0001	4765.6	4495.1 to 5036.1	< 0.0001	4801.0	4376.7 to 5225.4	< 0.0001
Age	486.7	422.8 to 550.6	< 0.0001	515.5	460.0 to 570.9	< 0.0001	516.2	432.7 to 599.8	< 0.0001
Genotype	295.5	52.3 to 538.7	0.017	−800.1	−1033.7 to −566.5	< 0.0001	−1525.9	−2216.3 to −835.6	< 0.0001
Age × genotype	−17.5	−68.3 to 33.3	0.500	−54.6	−101.3 to −8.0	0.022	−42.8	−175.2 to 89.7	0.527

POase									
Intercept	173.0	144.1 to 202.0	< 0.0001	547.1	499.2 to 595.1	< 0.0001	759.8	672.1 to 847.6	< 0.0001
Age	21.8	15.3 to 28.3	< 0.0001	94.7	81.0 to 108.5	< 0.0001	135.3	115.0 to 155.7	< 0.0001
Genotype	258.2	224.5 to 291.8	< 0.0001	−127.3	−164.6 to −89.9	< 0.0001	−627.4	−720.4 to −534.5	< 0.0001
Age × genotype	48.7	41.2 to 56.3	< 0.0001	−27.1	−37.8 to −16.5	< 0.0001	−112.6	−133.8 to −91.3	< 0.0001

a *PON1_192_* was coded 0, 1, and 2 for QQ, QR, and RR genotypes, respectively. The total number of children included in this model was 443.

b *PON1_-108_* was coded 0, 1, and 2 for CC, CT, and TT genotypes, respectively. The total number of children included in this model was 439.

c Individuals with the *PON1_192RR_* and *PON1_-108CC_* diplotype were coded 0, and individuals with the *PON1_192QQ_* and *PON1_-108TT_* diplotype were coded 1. The number of children included in this model was 84.

d Age was expressed in years.

e Age × genotype refers to the interaction term between age and genotype (*PON1_192_*, *PON1_-108_*, or *PON1_192–108_* diplotype).

**Table 4:** Regression analyses of age, genotype, and storage on PON1 enzyme activity in smaller age intervals.

	1 vs. 2 years of age^a^			5 vs. 7 years of age^b^			5 vs. 7 years of age, interaction model		
	β-Estimate	95% CI	*p*-Value	β-Estimate	95% CI	*p*-Value	β-Estimate	95% CI	*p*-Value
AREase									
Intercept	42.3	4.7 to 79.9	0.027	21.5	−28.1 to 71.0	0.396	23.2	−30.1 to 76.5	0.39
Age^c^	21.0	11.8 to 30.2	< 0.001	14.9	8.2 to 21.6	< 0.001	14.6	7.1 to 22.1	< 0.001
*PON1*_192_^d^	−4.8	−8.8 to −0.7	0.022	−5.3	−9.0 to −1.7	0.004	−7.3	−27.9 to 13.2	0.48
Storage duration^e^	1.4	−4.4 to 7.1	0.643	−3.9	−12.8 to 4.9	0.384	−3.9	−12.7 to 5.0	0.39
Age × *PON1*_192_^f^							0.3	−3.1 to 3.7	0.85

CPOase									
Intercept	3197.9	879.9 to 5515.9	0.007	1837.6	−985.3 to 4660.5	0.202	2356.9	−636.1 to 5349.9	0.12
Age	1288.9	712.0 to 1865.9	< 0.001	703.0	318.5 to 1087.5	< 0.001	617.4	197.9 to 1,036.9	< 0.001
*PON1*_192_	190.0	−58.2 to 438.3	0.133	188.5	−13.6 to 390.5	0.068	−412.0	−1644.7 to 820.7	0.51
Storage duration	−139.5	−492.2 to 213.3	0.438	−29.4	−542.0 to 483.2	0.911	−17.4	−530.5 to 495.8	0.95
Age × *PON1*_192_							96.7	−100.4 to 293.8	0.34

POase									
Intercept	280.1	−80.4 to 640.6	0.128	−133.3	−528.7 to 262.0	0.509	76.1	−333.2 to 485.4	0.72
Age	128.0	23.6 to 232.3	0.016	67.1	13.8 to 120.5	0.014	32.6	−23.2 to 88.4	0.25
*PON1*_192_	412.9	366.6 to 459.2	< 0.001	535.3	501.6 to 568.9	< 0.001	293.1	86.4 to 499.8	0.01
Storage duration	−55.8	−109.0 to −2.6	0.04	6.8	−65.3 to 79.0	0.853	11.7	−59.9 to 83.2	0.75
Age × *PON1*_192_							39.0	6.7 to 71.3	0.02

a This model included 52 children 1 year of age and 249 children 2 years of age. Of these children, 35 had measurements at both time points.

b This model included 225 children 5 years of age and 281 children 7 years of age. Of these children, 189 had measurements at both time points.

c Age was expressed in years.

d *PON1*_192_ was coded 0, 1, and 2 for QQ, QR, and RR genotypes, respectively.

e Storage duration was expressed in years.

f Age × *PON1*_192_ refers to the interaction term between age and *PON1*_192_ genotype.
